# Use of Traditional Japanese Herbal Medicine Daikenchuto for the Treatment of Abdominal Distention in Very-Low-Birth-Weight Infants

**DOI:** 10.3390/jcm13175082

**Published:** 2024-08-27

**Authors:** Shigeo Iijima

**Affiliations:** Department of Regional Neonatal-Perinatal Medicine, Hamamatsu University School of Medicine, Hamamatsu 431-3192, Japan; siijima@hama-med.ac.jp; Tel.: +81-53-435-2312

**Keywords:** herbal medicine, daikenchuto, very low birth weight infant, abdominal distention, abdominal radiography, gas volume score

## Abstract

**Background:** Very-low-birth-weight (VLBW) infants often experience feeding intolerance owing to organ immaturity, and the most frequent sign is the presence of abdominal distention. Daikenchuto (DKT), a traditional Japanese herbal medicine, is used to improve gastrointestinal function, particularly in adults. The aim of this study was to investigate the effectiveness of DKT in reducing abdominal distention and intestinal gas in VLBW infants. **Methods:** This study involved a retrospective chart review of 24 VLBW infants treated with DKT at Hamamatsu University Hospital between April 2016 and March 2021. The effects of DKT treatment at a dose of 0.3 g/kg/day were evaluated through clinical parameters and abdominal radiography. **Results:** Before treatment, marked abdominal distention was observed in 46% of the infants, which reduced to 4% within a week of DKT administration. The gas volume score (GVS) decreased in 92% of the patients within the first week of treatment and markedly decreased by ≥20% in 46% of the patients. The effects of improving abdominal distention and decreasing the GVS on radiography persisted for 1–2 weeks after treatment initiation. No clinical parameters affecting a GVS reduction of ≥20% and no notable adverse effects were observed. **Conclusions:** While the preliminary findings suggest that DKT may help manage abdominal distention in VLBW infants, further studies with placebo-controlled trials, larger sample sizes, use of advanced image processing software, and consideration of additional influencing factors are required to substantiate these results and identify predictors of treatment response.

## 1. Introduction

As very-low-birth-weight (VLBW) infants are at higher risk for health complications owing to organ immaturity, enteral nutrition in this population is challenging [1,2]. Fears of suspected feeding intolerance and the potential risk of necrotizing enterocolitis (NEC) frequently lead to delayed introduction of enteral feeding, slow advancement of feed volumes, and insufficient nutrient intake [3]. Abdominal distention, often caused by the accumulation of intestinal gas, is one of the most frequent signs of feeding intolerance in premature infants. Effective management of this condition is crucial for improving the outcomes in these infants [4].

Prokinetic agents have been used to improve feeding tolerance in VLBW infants. Erythromycin is a macrolide antibiotic that acts as a motilin receptor agonist and stimulates intestinal peristalsis [5]. However, erythromycin is associated with pyloric stenosis in early infancy [6,7] and QT interval prolongation in some patients [8,9]. An alternative prokinetic drug, metoclopramide, is a dopamine receptor antagonist used to promote gastric emptying [10] and to increase lower esophageal sphincter tone [11]. However, it is associated with serious adverse events related to dopaminergic dysregulation in patients of all ages [12].

Herbal medicines have been used in Japan for nearly 1500 years. Daikenchuto (DKT) is a traditional Kampo medicine composed of ginseng, processed ginger, and Japanese pepper with maltose [13]. DKT has been used for centuries to improve gastrointestinal (GI) function. DKT may promote intestinal motility and gas expulsion [14], and its clinical efficacy is now well established in adults and older children [15,16,17]. However, its efficacy in VLBW infants has not been extensively studied. This study aimed to evaluate the effects of DKT on intestinal gas volume in VLBW infants by comparing abdominal radiographs obtained before and during DKT treatment.

## 2. Materials and Methods

### 2.1. Patients and Data Collection

A retrospective chart review was conducted on consecutive VLBW infants who were born at the Perinatal Center of Hamamatsu University Hospital, admitted to the neonatal intensive care unit (NICU), and administered DKT during hospitalization between 1 April 2016 and 31 March 2021. The exclusion criteria were missing data, congenital malformations, metabolic disorders, cyanotic heart disease, sepsis, NEC, obstructive GI disease, and recent GI surgeries. In selected VLBW infants, clinical information including the degree of abdominal distention and intestinal gas volume on radiographs was compared before, within 1 week after, and 1–2 weeks after the start of DKT administration.

The collected maternal data included complications during pregnancy and conditions surrounding delivery, such as premature rupture of membranes (PROM), chorioamnionitis (CAM), pregnancy-induced hypertension (PIH), gestational diabetes mellitus (GDM), and antenatal steroid use. The attending obstetrician clinically diagnosed PROM, CAM, and PIH. Antenatal steroid use was defined as maternal administration of one or more doses of betamethasone. The infant data collected included gestational age (GA), birth weight (BW), sex, Apgar score, respiratory distress syndrome (RDS), patent ductus arteriosus (PDA), and intraventricular hemorrhage (IVH). The GA at birth was determined by maternal history based on the last menstrual period and obstetric examination with ultrasonography. Apgar scores were assessed at 1 min and 5 min. RDS was diagnosed based on clinical and chest radiographic findings and was listed if surfactant replacement treatment was required. PDA was diagnosed if indomethacin therapy was required and initiated when significant PDA was documented on serial echocardiography. IVH was diagnosed based on transfontanelle ultrasound findings. The postnatal age at the first and last DKT exposure and the duration of therapy were determined. The treatment duration was defined as the total number of days that each infant was exposed to DKT.

### 2.2. Nutritional Policy for VLBW Infants

Parenteral and enteral nutrition were prescribed daily in the NICU. Enteral feeding with non-fortified human milk was started after 24 h of age at a volume of 0.5–1.0 mL/kg/dose every 3 h if their respiratory and cardiovascular status were stable. Human milk feeding was encouraged, and the formula was fed if human milk was unavailable. Daily increases in enteral nutrition were 0.5–1.0 mL/kg/dose in neonates weighing < 1000 g and 1.0–2.0 mL/kg/dose in neonates weighing < 1500 g in the absence of feeding intolerance suspecting NEC. Fortifiers (HMS-2^®^; Morinaga Milk Industry, Tokyo, Japan) were added after enteral nutrition had reached a minimal volume of 50 mL/kg/day; fortifier was initiated at one-quarter concentration, raised to half concentration in 2–3 days, and raised to the normal dose (1.3 g HMS-2^®^/30 mL breast milk) in 2–3 days. Parenteral nutrition was discontinued, and the central venous catheter was withdrawn once enteral nutrition was well tolerated at a volume of 100–120 mL/kg/day. Enteral nutrition was administered continuously using a nasogastric tube until the weight was 1500 g, and the corrected GA was 35 weeks. Prefeed aspiration was systematically performed, and gastric residuals (GRs) were assessed before each feeding. Every 3 h, the nurses applied gentle massage to the infants’ abdomen to ensure that the tip of the nasogastric tube did not adhere to the wall of the stomach while the GRs were aspirated. Enemas (25% glycerin, 1–2 mL/kg/dose) were occasionally administered if no transit was observed for 24 h during the 48 h after birth. Subsequently, enemas were routinely administered thrice a day. If abdominal distention or delayed defecation was observed, enemas were administered six times a day to accelerate the passage of the meconium.

### 2.3. DKT Administration

DKT (15 g) (Tsumura & CO., Tokyo, Japan) has been used to treat intestinal dysmotility owing to paralytic ileus in adult patients at a daily dosage of 15 g orally in two or three divided doses before or between meals [18]. In this study, DKT was administered to VLBW infants at a dose of 0.3 g/kg/day. The daily dosage of DKT was evenly divided into three portions and dissolved in distilled lukewarm water. The supernatant liquid was administered into the stomach through a nasogastric tube every 8 h.

### 2.4. Evaluation of Clinical Parameters and Manifestations

The evaluations performed before and during DKT treatment included the number of defecations and glycerin enemas used per day, total amount of GRs per day, and degree of abdominal distention. The degree of abdominal distention was graded as none (grade 0), mild (grade 1), moderate (grade 2), or marked (grade 3) based on visual examination by the attending nurse.

### 2.5. Evaluation of Abdominal Gas Areas

**Figure 1:**
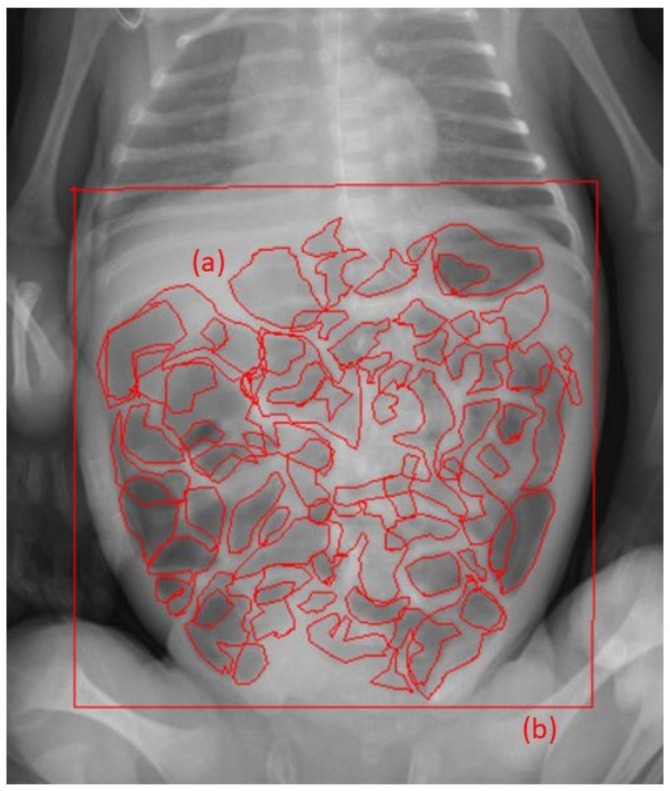
Estimation of gas volume score (GVS) in a plain abdominal radiograph. The outlines of the intestinal gas were manually traced, and the area was calculated by counting the pixel value using ImgWorks; all areas were summed in (**a**). The pixel count of the rectangular area (window of the abdominal area) is calculated in (**b**). GVS calculated as (**a**)/(**b**) of this image is “46,176.5/97,968 = 0.471”.

Plain abdominal radiographs of the patients in the supine position recorded in the morning (at 9 a.m.; before feeding) before and during DKT treatment were evaluated. The radiographs were digitized and transmitted to a computer. The region of abdominal gas was distinguished from the other portions by increasing the contrast. To determine the total abdominal gas area in a radiographic image, the region of abdominal gas on the digital image was identified, the outline of the abdominal gas was manually traced (Figure 1), the area was calculated by counting the pixel values, and all areas were summed. ImgWorks, a free software developed by Tamanomori, was used to measure the pixel values [19]. The gas volume score (GVS) was defined as the entire area of the traced abdominal gas divided by the area surrounded by a horizontal line tangential to the infra-ilium margin, a horizontal line tangential to the uppermost diaphragm, and the most lateral line tangential to the right and left abdominal walls; this is a modification of Koide’s method [20] that can be applied to neonates. All measurements and calculations of abdominal gas areas and GVSs were performed by a single operator (the corresponding author).

### 2.6. Evaluation of Adverse Effects

Drug safety was evaluated by determining the incidence of adverse events for laboratory and clinical parameters. The candidates for possible side effects were determined based on a previous study by Katori et al. [21]. Serum aspartate transaminase, alanine transaminase, blood urea nitrogen, and creatinine levels were evaluated before and after the DKT treatment. Clinical adverse effects that occurred when the infant was exposed to the medication of interest included rash, seizure, arrhythmia, focal intestinal perforation, surgical or NEC, pyloric stenosis, and IVH. Additionally, GI discomfort was evaluated through clinical observations including signs of irritability and aggravation of feeding tolerance, stool characteristics, and abdominal distension. Death was defined as death before discharge from the NICU. The observation period for potential adverse effects extended throughout the infants’ stay in the NICU and for an additional follow-up period of 6 months post discharge. During the post-discharge follow-up period, readmissions were tracked for GI symptoms, liver dysfunction, and interstitial pneumonia.

### 2.7. Statistical Analyses

The data obtained during this study were analyzed using the Statistical Package for Social Sciences (version 25; IBM Corporation, Armonk, NY, USA). Data are presented as median with interquartile range (IQR) for continuous variables and as number and percentage for categorical variables to describe the study variables. Non-parametric methods (Spearman’s correlation coefficient, Kruskal–Wallis test, and Mann–Whitney U test) were used, where appropriate, to assess the influence of DKT administration. All statistical tests were two-sided, and a *p* < 0.05 was considered statistically significant.

### 2.8. Ethics Approval

The present study was designed and conducted in accordance with the principles outlined in the Declaration of Helsinki and approved by the Ethics Committee of Hamamatsu University School of Medicine (approval number: 21–260). For this retrospective study, the patients’ parents were not required to provide informed consent because the analysis used anonymous clinical data that had been obtained after each patient’s parent provided written consent for clinical management. Moreover, an opt-out method was provided for obtaining consent on our hospital’s website.

The administration of DKT to VLBW infants in this study was conducted in accordance with the Japanese medical practices and regulations. As DKT is permitted for use in neonates in Japan, specific ethics committee approval and parental consent were not required for its administration in this patient population. However, when administering DKT, the attending physician fully explained the medical condition and the drug to the parents and obtained their consent to administer the drug. In addition, the medical team provided comprehensive care and support to all parents throughout the treatment process, ensuring that their overall experience was as supportive and understanding as possible.

## 3. Results

### 3.1. Samples

During the study period, 175 VLBW infants were admitted to the NICU in Hamamatsu University Hospital, and 24 infants (17 male and 7 female infants) were treated with DKT for abdominal distention during hospitalization in the study period.

The infants had a median GA of 26.4 (IQR, 24.8–29.4) weeks and a median BW of 808 (IQR, 577–1161) g. The profiles were as follows: 67% with PROM; 75% CAM; 17% PIH; 4% GDM; 25% antenatal steroid use; 1 min Apgar score of 3 (IQR, 1–5) and 5 min Apgar score of 7 (IQR, 6–7); 79% with RDS; 71% PDA; and 4% IVH. The median age at the time of initial DKT administration was 17 (IQR, 9–42), the median age at the end of DKT administration was 94 (IQR, 53–129), and the median duration of exposure to DKT was 61 (IQR, 36–97) days.

### 3.2. Changes in Clinical Data and GVS

**Figure 2:**
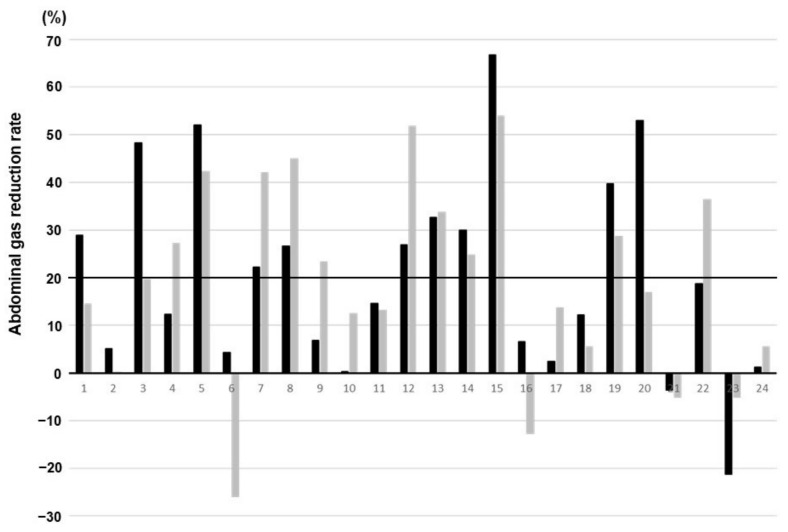
The reduction rate of gas volume score (GVS) in individual cases. Black bars indicate radiographs recorded within 1 week after the start of daikenchuto administration, and gray bars indicate radiographs recorded at 1–2 weeks after the start of drug administration. The black line at 20% represents the threshold used to categorize patients into two groups: those with a GVS reduction of ≥20% within 1 week and those with less than 20%.

**Figure 3:**
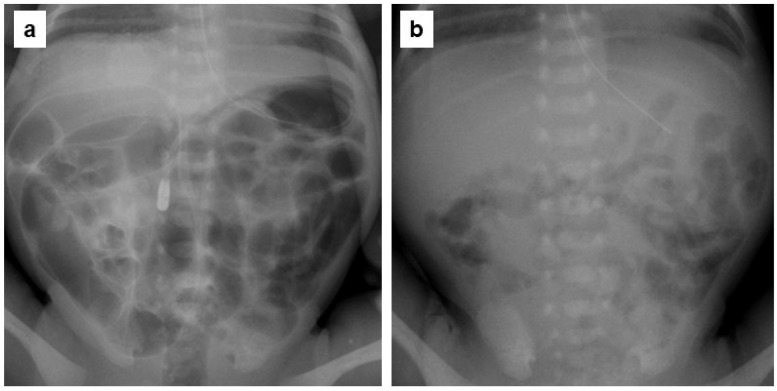
Plain abdominal radiographs of a very-low-birth-weight infant showing a marked daikenchuto (DKT) effect in the same patient. (**a**) Seven days after birth (2 days prior to DKT administration). The gas volume score (GVS) was calculated as 0.554. (**b**) Twelve days after birth (3 days after the start of DKT therapy). The GVS was calculated as 0.184.

A comparison of clinical parameters, GVS, and GVS reduction effect on abdominal radiographs before and within 1 week after the initiation of DKT administration and 1–2 weeks of treatment is shown in Table 1. Abdominal distention was 46% marked, 42% moderate, and 12% mild before treatment, but within 1 week after the initiation of treatment, it was 4% marked, 79% moderate, and 17% mild. Within 1–2 weeks after the initiation of treatment, it was 4% marked, 71% moderate, and 25% mild, showing statistically significant improvement (*p* = 0.008). The GVS decreased in 92% of the patients within 1 week and in 83% of the patients after 1–2 weeks of treatment with DKT compared with that before treatment. The GVS was significantly positively correlated with the degree of abdominal distention (grades 0, 1, 2, and 3) (r = 0.52, *p* < 0.001). The percentage of GVS reduction in the individual cases is shown in Figure 2. For convenience, a decrease of ≥20% was considered a significant decrease in GVS, and 46% of the patients showed a significant decrease in GVS within 1 week and 1–2 weeks of treatment (Table 1). Representative abdominal radiographs of the same patient before and after DKT administration showed reduced intestinal gas volumes (Figure 3a,b). In this case, DKT administration reduced the GVS from 0.554 to 0.184 (reduction rate, 67%). Next, clinical parameters were compared between the 11 patients who showed a GVS reduction of ≥20% on radiographs within 1 week after the start of DKT administration and the other patients, and no significant differences were found in all parameters, including the degree of abdominal distention (Table 2).

### 3.3. Adverse Effects

Clinical adverse effects were monitored during and after DKT administration. No significant adverse effects such as rash, seizure, arrhythmia, focal intestinal perforation, NEC, pyloric stenosis, IVH, GI discomfort (signs of irritability and aggravation of feeding tolerance, stool characteristics, and abdominal distention), death before NICU discharge, or abnormalities in blood biochemistry (Table 3) were observed. The observation period for adverse effects extended to 6 months post discharge, and readmissions due to GI symptoms, liver dysfunction, and interstitial pneumonia were not observed.

## 4. Discussion

This study investigated the effects of DKT on abdominal distention in VLBW infants by comparing intestinal gas volume before and during DKT treatment. The results indicated a trend toward improvement in abdominal distention and a reduction in intestinal gas volume following DKT administration. These findings suggest that DKT may be effective in managing abdominal distention in VLBW infants.

In premature infants, feeding intolerance is extremely common and is often evidenced by abdominal distention. Abdominal distention, identified through physical examination and radiography showing dilated gas-filled loops of the bowel, involves trapped gas, abdominal pressure, and fullness. The causes include feeding intolerance, infections that disrupt the gut microbiota, delayed intestinal transit, and abnormal reflux of visceral fluid [22].

DKT effectively treats abdominal distention [23,24,25,26,27]. Animal models have suggested that DKT promotes GI motility through elevated levels of plasma vasoactive intestinal polypeptide [28], substance P [28,29], motilin [30], and acetylcholine [31] and increases intestinal blood flow via calcitonin gene-related peptide and adrenomedullin [29,32]. The anti-inflammatory effects of DKT [14,33] and its antagonistic effects on 5-hydroxytryptamine receptor 3A receptors [34] may improve visceral hypersensitivity. DKT reduces intestinal gas volume. Clinical studies have shown a significant decrease in the intestinal gas in patients treated with DKT, suggesting its efficacy in the management of excessive intestinal gas [23,24,35]. DKT enhances GI motility and reduces gas buildup by promoting the effective transit of intestinal contents [35]. Additionally, DKT positively influences the gut microbiota, increasing beneficial bacterial populations and microbial metabolites such as short-chain fatty acids, which reduce gas production and improve gut health [35]. However, further studies with untreated control groups are required to confirm its efficacy and mechanism of action.

Several studies have investigated the GI benefits of DKT in neonates. Watanabe et al. found that DKT improved blood flow in the superior mesenteric artery and portal vein in extremely-low-birth-weight infants [36]. Shinyama et al. observed that DKT reduced the incidence and severity of NEC in a rat model by enhancing GI motility, suppressing bacterial overgrowth, increasing bowel blood flow, and reducing inflammation [37]. Despite these findings, no studies have specifically examined the effects of DKT on abdominal distention or gas production in neonates. This study is the first to report the clinical efficacy of DKT in VLBW infants with abdominal distention, suggesting its potential as a therapeutic option for neonatal care.

The reported incidence rate of adverse effects associated with DKT, such as GI discomfort, liver dysfunction, and interstitial pneumonitis, is 2.0% [21]. However, the safety of DKT in neonates has not been established due to insufficient clinical data. In this study, no adverse effects were observed during and after DKT administration and for an additional follow-up period of 6 months post discharge. Therefore, the treatment is generally considered safe, even in VLBW infants. According to animal reproductive toxicology studies, DKT has failed to pose a risk to the fetus. Tsuda et al. investigated the efficacy and safety of DKT in pregnant women with constipation and found that the clinical course of mothers and fetuses during DKT treatment was uneventful [38].

This study has several strengths. This is the first study to evaluate the efficacy of DKT in managing abdominal distention in VLBW infants. Moreover, this is the first quantitative evaluation of intestinal gas using radiography in the neonatal field. The use of plain abdominal radiography to evaluate the GVS quantitatively provides objective and reproducible data. This method involves digitizing radiographs and using software to measure the area of abdominal gas and quantitatively evaluating GVS, providing an objective and quantifiable metric for gas volume. This is more precise than the visual estimation alone.

Despite these potential results observed in this study, these findings should be interpreted with caution. Several limitations must be addressed. First, this study was conducted with a relatively small sample size of 24 infants, limiting the generalizability of the results. Therefore, large-scale studies are required. Second, the absence of a control group meant that the potential influence of placebo effects or natural improvements over time could not be ruled out. Future studies should include placebo-controlled trials, larger sample sizes, and the consideration of other factors that may influence the child’s health status, such as type of food fed, use of glycerin enema, and maturity. These measures will help confirm the efficacy of DKT and identify reliable predictors of treatment response. Third, a selection bias could have occurred in selecting the study participants because the DKT-dosing criterion was not established, and the decision to administer DKT was at the attending neonatologist’s discretion. Next, the lack of significant differences in clinical information, including the degree of abdominal distention between those with significant GVS reduction and those without, indicates that an abdominal distention scoring system for objective evaluation and detailed examination of other potential influencing factors (e.g., nutritional status and concomitant medications) is lacking. In addition, limitations exist for the methodology used for evaluating abdominal distention and gas. The initial grading of abdominal distention based on visual examination by an attending nurse is highly subjective and can vary significantly among nurses. This introduces variability and a potential bias. Manual tracing of the outline of the abdominal gas and counting of pixels are labor-intensive and prone to human error. This can lead to inconsistencies, especially if only one operator performs all measurements, as operator fatigue or bias can affect the results. Moreover, free software can vary in quality. Without validation, its suitability for precise medical measurements is uncertain. The use of advanced image processing software for automated measurements of intestinal gas volume is recommended to reduce manual effort and potential errors.

## 5. Conclusions

DKT shows potential in reducing abdominal distention and intestinal gas in VLBW infants. While this study suggests that DKT may be an effective complementary therapy, the results are preliminary and require further validation. Future studies should focus on identifying reliable predictors of treatment response, optimizing dosage and administration duration, and conducting placebo-controlled trials with larger sample sizes, use of advanced image processing software, and consideration of additional influencing factors to provide more robust evidence on the efficacy of DKT. This study provides a valuable foundation for future investigations and highlights the potential clinical benefits of integrating traditional medicine into modern neonatal care.

## Figures and Tables

**Table 1:** Comparison of clinical parameters and gas volume scores based on abdominal radiographs before and during daikenchuto treatment.

	Before Treatment	≤1 Week of Treatment	1–2 Weeks of Treatment	*p*-Value
Age, day after birth	16 (6.3–42)	21 (12–45)	30 (21–57)	0.023
Days after the start of treatment, days	-	3 (2–4)	14 (9–16)	<0.001
Mechanical ventilation, *n* (%)	21 (88)	20 (83)	15 (63)	0.086
Feeding volume, mL/day	104 (29–138)	123 (74–142)	139 (127–148)	0.004
Degree of abdominal distention, grade 0:1:2:3 *	0:3:10:11	0:4:19:1	0:6:17:1	0.004
Frequency of defecation, /day	4 (3–6)	6 (3–6)	6 (5–6)	0.10
Glycerin enema frequency, /day	5 (3–6)	6 (3–6)	6 (3–6)	0.49
Gastric residual volume, mL/day	5 (4–8)	4 (3–6)	4.5 (0.5–5.8)	0.14
Gas volume score (GVS)	0.40 (0.31–0.49)	0.30 (0.24–0.38)	0.31 (0.23–0.35)	0.008
Reduction of GVS, *n* (%)	-	22 (92)	20 (83)	0.39
≥20% reduction of GVS, *n* (%)	-	11 (46)	11 (46)	1.0

Categorical variables are expressed as number (%). Continuous variables are expressed as median (interquartile range). * The degree of abdominal distention was graded as none (grade 0), mild (grade 1), moderate (grade 2), or marked (grade 3).

**Table 2:** Relationship between intestinal gas reduction and clinical parameters within the first week of daikenchuto treatment.

	Reduction Rate ≥ 20% (*n* = 11)	Reduction Rate < 20% (*n* = 13)	*p*-Value
Sex (male), *n* (%)	8 (73)	9 (69)	0.91
Gestational age, weeks	27.7 (25.3–30.6)	26.4 (24.5–29.2)	0.46
Birth weight, g	846 (558–1166)	760 (620–1171)	0.73
Premature rupture of membranes, *n* (%)	3 (27)	5 (38)	0.65
Chorioamnionitis, *n* (%)	2 (18)	4 (31)	0.61
Pregnancy-induced hypertension, *n* (%)	3 (27)	1 (8)	0.42
Gestational diabetes mellitus, *n* (%)	1 (9)	0 (0)	0.73
Antenatal steroid use, *n* (%)	3 (27)	3 (23)	0.87
1 min Apgar score	3 (1–4)	3 (1–5)	0.87
5 min Apgar score	6 (5–8)	7 (6–7)	0.65
Respiratory distress syndrome, *n* (%)	9 (82)	10 (77)	0.87
Patent ductus arteriosus, *n* (%)	7 (64)	10 (77)	0.61
Intraventricular hemorrhage, *n* (%)	1 (9)	0 (0)	0.73
Age at start of enteral feeding, day after birth	3 (1–3)	2 (2–3)	1.00
Age at time of full enteral feeding, day after birth	15 (12–29)	14 (12–19)	0.96
Age at start of treatment, day after birth	28(15–42)	10 (8–51)	0.33
Age at end of treatment, day after birth	113 (45–130)	75 (64–138)	0.73
Treatment period, days	48 (36–101)	62 (42–98)	0.69
At time of pre-treatment radiography			
Mechanical ventilation, *n* (%)	8 (73)	12 (92)	0.42
Age, day after birth	28 (15–42)	8 (5–44)	0.19
Feeding volume, mL/day	110 (100–140)	88 (20–120)	0.17
Degree of abdominal distention, grade 0:1:2:3 *	0:0:4:7	0:3:6:4	0.09
Frequency of defecation, /day	6 (3–6)	3 (3–6)	0.13
Glycerin enema frequency, /day	6 (3–6)	3 (3–6)	0.07
Gastric residual volume, mL/day	4 (4–8)	5 (4–9)	0.65
At radiography within 1 week of initiation of treatment			
Mechanical ventilation, *n* (%)	8 (73)	12 (92)	0.42
Age, day after birth	30 (18–45)	15 (10–53)	0.39
Days after the start of treatment, days	3 (2–3)	3 (2–5)	0.87
Feeding volume, mL/day	135 (120–143)	116 (68–136)	0.28
Degree of abdominal distention, grade 0:1:2:3 *	0:0:11:0	0:4:8:1	0.36
Frequency of defecation, /day	6 (6–6)	5 (3–6)	0.21
Glycerin enema frequency, /day	6 (6–6)	6 (3–6)	0.23
Gastric residual volume, mL/day	4 (0–6)	5 (4–8)	0.25

Categorical variables are expressed as number (%). Continuous variables are expressed as median (interquartile range). * The degree of abdominal distention was graded as none (grade 0), mild (grade 1), moderate (grade 2), or marked (grade 3).

**Table 3:** Biochemical parameters before, during, and after daikenchuto treatment.

Parameter	Pre-Treatment	Under Treatment		After Treatment (Pre-Discharge)	Reference Range
		1 Week *	2 Weeks *		
Blood urea nitrogen (mg/dL)	10.3 (2.6–13.0)	6.0 (2.0–13.2)	4.1 (2.3–11.0)	3.8 (2.1–10.3)	4–20
Creatinine (mg/dL)	0.55 (0.20–0.90)	0.44 (0.17–0.77)	0.38 (0.16–0.71)	0.22 (0.13–0.38)	0.2–0.9
Aspartate transaminase (U/L)	30.5 (17–45)	29.5 (19–44)	30.5 (21–45)	34.5 (24–44)	11–59
Alanine transaminase (U/L)	7.5 (3–32)	7.5 (2–31)	7.5 (5–26)	14.0 (6–26)	4–30

Variables are expressed as median (minimum–maximum). * Time from the start of treatment to examination.

## Data Availability

The original contributions presented in the study are included in the article; further inquiries can be directed to the corresponding author.
